# Non-overlapping sets of neurons encode behavioral response determinants across different tasks in the posterior medial prefrontal cortex

**DOI:** 10.3389/fnsys.2023.1049062

**Published:** 2023-02-09

**Authors:** Muhammad Ali Haider Awan, Hajime Mushiake, Yoshiya Matsuzaka

**Affiliations:** ^1^Laboratory of System Neuroscience, Department of Physiology, Tohoku University, Sendai, Japan; ^2^Division of Neuroscience, Faculty of Medicine, Tohoku Medical and Pharmaceutical University, Sendai, Japan

**Keywords:** posterior medial prefrontal cortex, task-dependent encoding, behavioral tactics, neuron, monkey

## Abstract

Higher mammals are able to simultaneously learn and perform a wide array of complex behaviors, which raises questions about how the neural representations of multiple tasks coexist within the same neural network. Do neurons play invariant roles across different tasks? Alternatively, do the same neurons play different roles in different tasks? To address these questions, we examined neuronal activity in the posterior medial prefrontal cortex of primates while they were performing two versions of arm-reaching tasks that required the selection of multiple behavioral tactics (i.e., the internal protocol of action selection), a critical requirement for the activation of this area. During the performance of these tasks, neurons in the pmPFC exhibited selective activity for the tactics, visuospatial information, action, or their combination. Surprisingly, in 82% of the tactics-selective neurons, the selective activity appeared in a particular task but not in both. Such task-specific neuronal representation appeared in 72% of the action-selective neurons. In addition, 95% of the neurons representing visuospatial information showed such activity exclusively in one task but not in both. Our findings indicate that the same neurons can play different roles across different tasks even though the tasks require common information, supporting the latter hypothesis.

## Introduction

Little is known about how the neural network enables flexible use of the same information for diverse purposes (e.g., a phone number to make a call or to use as a password). In other words, how the finite number of neurons in the brain gives rise to an infinite number of behaviors in a dynamic environment is unclear. Does a neuron subserving a particular role (e.g., spatial working memory) in one task also play the same role in different tasks? Alternatively, do neurons change their functions under different behavioral requirements? To address this issue, we examined neuronal activity from the dorsomedial prefrontal cortex of primates, especially its posterior part (pmPFC) while monkeys were switching between two variants of arm reaching tasks. The dorsomedial prefrontal cortex has been implicated in a wide array of functions, including mindfulness (Sezer et al., 2022), conflict reduction by biasing behavior (Nakao et al., 2010), as a comparator for different stimulus values (Hare et al., 2011), suppression of distractors or irrelevant options (Noonan et al., 2017), and predicting the behavior or intentions of others (Isoda and Noritake, 2013). These findings indicate that the neural circuits in this region can switch their functions to meet diverse behavioral requirements. In addition, functional neuroimaging studies demonstrated activation of the medial frontal cortex during task switching (Rushworth et al., 2002; Crone et al., 2006). However, little is understood about how the neural network in the medial prefrontal cortex switches the function across different tasks.

Previous studies have shown that, in the lateral prefrontal cortex (lPFC), the neuronal representation of relevant sensory stimuli varies across multiple tasks referred to by their usage for different purposes, e.g., a picture serving as a sample stimulus of a delayed matching to sample (DMS) task vs. a cue to call for a conditional motor response (Asaad et al., 2000; Warden and Miller, 2010). On the other hand, the prefrontal cortex has also been shown to process more abstract information (e.g., rules, categories, and quantities) inferred from sensory stimuli rather than mere physical properties of stimuli (Freedman et al., 2001; Wallis et al., 2001; Miller et al., 2002; Nieder et al., 2002; Nieder and Miller, 2003). However, it remains unclear how the prefrontal cortex uses such stimulus-derived information for multiple tasks.

Recently, we reported that the primate posterior medial prefrontal cortex (pmPFC) participates in the selection of behavioral tactics (Matsuzaka et al., 2012, 2016; Awan et al., 2020). It is an internal protocol of how to decide what to do, and therefore the selection of tactics differs from the selection of the action *per se*, which is decision-making of what to do. These studies, however, examined neuronal activity during the performance of a single version of an arm reaching task. Therefore, it remained unclear how the neural representation of a stimulus, tactics inferred from stimuli, and the final action would change across different conditions.

To address this issue, we examined neuronal activity in the pmPFC during the performance of two variants of tactics-based action selection tasks. Both tasks required the monkey to integrate the response tactics with the spatial information to determine the action but the temporal order of presentation of tactics cue and the spatial cue was different. We found that the majority of neurons in the pmPFC encoded response determinants (either tactics or spatial position) or resultant actions in one task paradigm, but changed their representation in the other task. Our findings indicated that the same function can be subserved by different groups of neurons even though the tasks require common sets of information relevant to the task performance.

## Materials and methods

### Experimental setup and behavioral task

**Figure 1:**
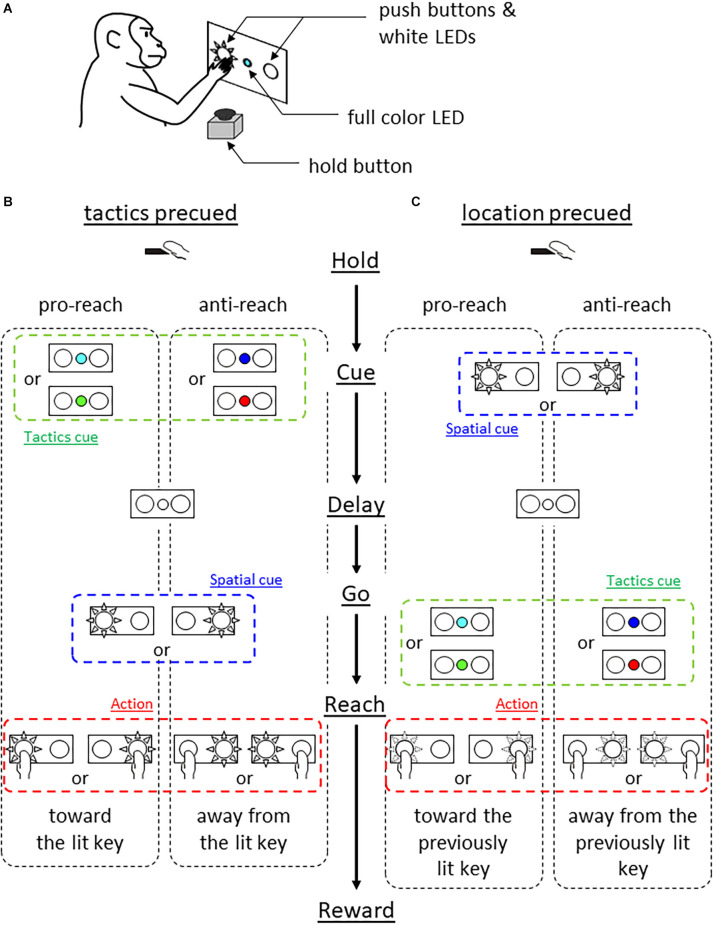
Behavioral experiment. **(A)** Experimental setup. Monkey faced a panel equipped with a full color LED in the center and two push buttons which were back-illuminated by white LEDs. **(B)** *Tactics-precued task*. Following a hold period of 1 s, the center LED was turned on in one of the four colors for 500 ms. The color cue instructed the tactics (cyan and green for pro-reach, blue and red for anti-reach). After variable delay period (1–1.5 s, either the left or right push button was back-illuminated in white serving as the visuospatial cue and 1-kHz tone was simultaneously turned on, prompting the monkey’s response. Pro-reach trials required reaching toward the illuminated button while anti-reach required reaching away from the illuminated button. **(C)** *Location-precued task*. After the hold period, either the left or the right button was back-illuminated in white for 0.5 s serving as the visuospatial cue. A variable delay period (1–1.5 s) followed, then the center LED was turned on in one of the four colors, serving as the tactics cue simultaneously with the 1 kHz tone. The color-tactics association was the same as in the tactics-precued task. The monkey selected the appropriate action based on the remembered cue location and the given tactics.

We used a monkey (*Macaca fuscata*, male, weighing 10 kg), cared for in accordance with the guidelines of the National Bioresource Project of Japan and our institutional guidelines. The monkey sat in a primate chair and faced a switch panel (Figure 1A). The panel housed a full color light emitting diode (LED) in its center and two push buttons, one on the left and the other on the right, which were back-illuminated by white LEDs.

The monkey was trained to press either the left or the right button under two different conditions. Both tasks required the monkey to integrate visuospatial information (either left or right) and response tactics (either reach to or away from the spatial cue) to determine the action (reach to either the left or the right target). A trial began when the monkey pressed the hold button attached to the armrest of the primate chair for 1 s. In the *tactics-precued* *task* (Figure 1B), the center LED was turned on for 0.5 s in one of the four colors to indicate the tactics of the forthcoming reach. The cyan and green color instructed the monkey to reach toward the subsequent visuospatial cue (pro-reach) whereas the blue and red colors required the reach away from the visuospatial cue (anti-reach). After the variable length of delay period (1–1.5 s), either the left or the right push button of the panel was back-illuminated by a white LED, prompting the monkey’s response. The monkey was rewarded by pressing the illuminated button under the pro-reach condition, whereas it was rewarded by pressing the non-illuminated button under the anti-reach condition within 1 s.

In the *location-precued task* (Figure 1C), after the hold period, either the left or the right push button was illuminated in white for 0.5 s. After the delay period, the center LED was turned on in one of the four colors to instruct the tactics of the reach while prompting the monkey’s response. The association between the cue color and the tactics was the same as in the tactics-precued task. The monkey was able to learn the task (mean correct rate tactics pre-cued 83% and location pre-cued 81%).

### Neuronal recording

At the end of the training, the monkey underwent surgery to install a chamber that covered the pmPFC and the adjacent presupplementary and supplementary motor areas (pre-SMA and SMA, respectively). After the recovery period, we recorded neuronal activity with elgiloy electrodes (0.9–1.2 MΩ at 1 kHz). The electrodes were advanced by hydraulic manipulators (MO-81 of Narishige Inc., Tokyo, Japan). We identified unitary action potentials by the RASPUTIN software (Plexon Inc., Texas, USA) as distinct clusters in its feature space during the experiments as well as in offline spike-sorting after each experiment. Further, we visually inspected the waveforms in the identified clusters. If they were indistinguishable from baseline noise, such data were discarded. While advancing the electrodes, if we found any task-related neurons, to ensure the same neuron was recorded across different conditions, we presented the same behavioral condition twice separated by other behavioral conditions. The selection criteria for neurons were at least five correct trials in all possible combinations for tactics pre-cued and location pre-cued trials.

To define borders between the medial areas, in each electrode penetration, we examined neuronal responses to visual and somatosensory stimuli by illuminating the monkey’s eye with a flashlight, tapping on the monkey’s body surface, and manipulating the joints. We also examined evoked movements by applying intracortical microstimulation (cathodal current: 10–80 μA; pulse width: 300 μs; interval: 3 ms; 12–80 pulses) through the recording electrode.

### Statistical analysis

We wrote a software in C++ for offline display and quantitative analysis of neuronal activity. Statistical analysis was done using R. As behavioral response determinants, we defined tactics (pro- or anti-reach), cue position (left or right), and cue color (cyan, green, blue, or red). Additionally, we defined action as reaching either to the left or the right target. Moving time window analysis was performed to examine the temporal variance of neuronal representations of response determinants and action. In this analysis, we counted action potentials within the small time window (width 200 ms) to calculate the instantaneous firing rate (IFR) at time *t*. These windows included pre-cue, post-cue, delay, and response periods, and were shifted in step sizes of 20 ms to calculate the IFR(*t*) at each new position. We repeated this procedure to obtain the temporal change in IFR(*t*) for all epochs.

To examine the effects of the response determinants and the action on the neuronal activity, we performed a multivariate analysis of variance (ANOVA) using tactics, cue position, action, and cue color as factors and firing rate as the dependent variable. To quantify the selectivity of IFR(*t*) for response determinants and action, we computed the coefficient of partial determination (CPD). CPD(*X, t*) was the percentage of the variance of IFR(*t*) ascribable to the variance of the particular factor *X* (e.g., tactics) defined as::


$$CPD(X,t)\text{=}\frac{SSEpartial\;(t)-SSEfull\;(t)}{SSEpartial\;(t)}$$

where SSEpartial(*t*) was the sum of squared errors when factor *X* was omitted from the ANOVA and SSEfull(*t*) was the sum of squared errors when all the factors were included.

Finally, to examine how neurons change their representations of the response determinants and the action across the tasks, we analyzed their effects on the neuronal activity during the response period where all the information was available to the monkey in both tasks. For this purpose, we performed multifactorial ANOVA using the neuronal firing rate during the 300 ms interval preceding movement onset as the dependent variable, and tactics (pro-reach or anti-reach), action (left or right reach), and cue location (left or right) as factors.

## Results

We identified the pmPFC, SMA, and pre-SMA using previously reported criteria (Matsuzaka et al., 2012, 2016). The SMA was characterized by the topographically organized somatosensory receptive fields of the neurons, their lack of response to visual stimuli, and the relative ease of eliciting bodily movements by electrical stimulation. The pre-SMA, located anteriorly to the facial region of the SMA, had an abundance of visually responsive neurons, which responded poorly to tactile stimuli. Further, in the pre-SMA, electrical stimulation rarely evoked movement, and when evoked, their thresholds were higher than those in the SMA. Finally, the pmPFC, located anteriorly to the pre-SMA, was characterized by a lack of response to visual as well as tactile stimuli, and the absence of evoked movements by the strongest stimuli tested (Supplementary Figure S1).

We recorded a total of 213, 104, and 164 task-related neurons in the pmPFC, the pre-SMA, and the SMA of both hemispheres, respectively.

**Figure 2:**
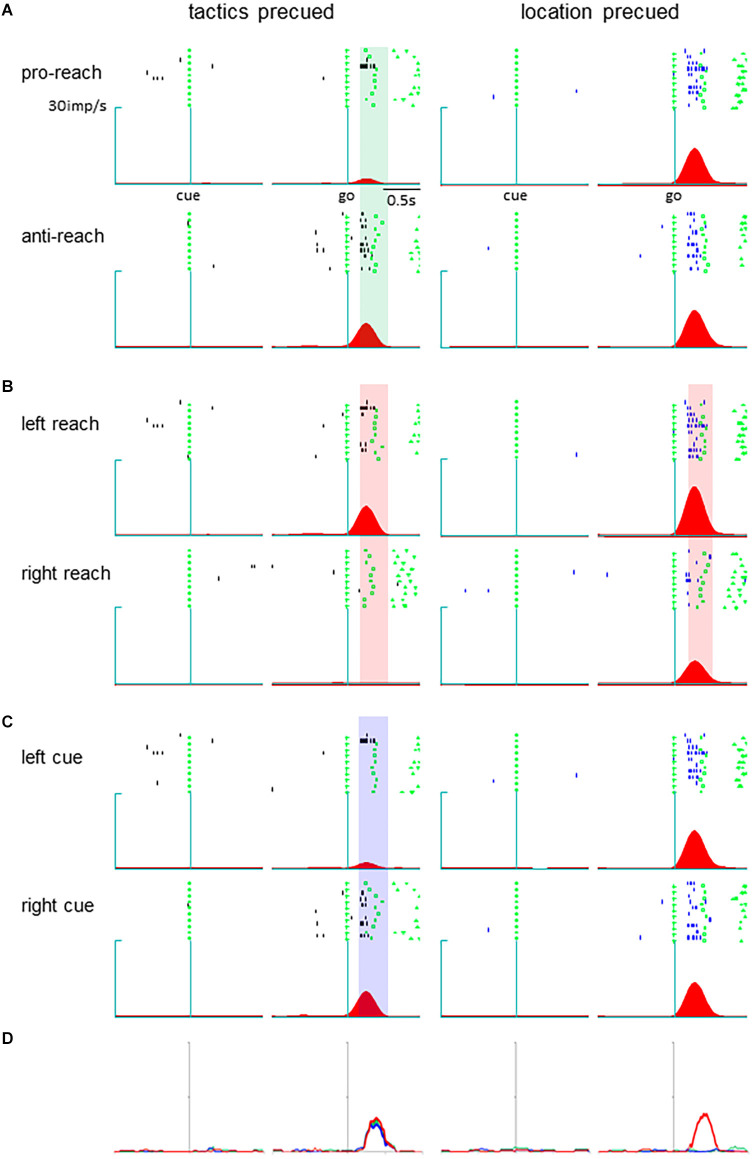
A pmPFC neuron encoding different information across different tasks during the delay period. **(A–C)** Raster display and spike density function of the neuronal activity. The trials are grouped by tactics **(A)**, action **(B)**, and cue location **(C)**. Activity during the tactics- and the location-precued tasks are shown in the left and the right columns, respectively. In each column, trials are aligned with cue onset (left) and go signal onset (right). The shaded areas are the time intervals where the tactics **(A)**, action **(B)**, and cue location **(C)** had significant effect on the neuronal activity. **(D)** The temporal variance of tactic selectivity (green color), action selectivity (red color), and cue location (blue color) illustrated as time-resolved change in the coefficient of partial determination (CPD) value. The thick line indicates significant effect of the respective factor on IFR(*t*) (*p* < 0.05 by ANOVA). *Left*: tactics-precued task, *right*: location pre-cued task.

**Figure 3:**
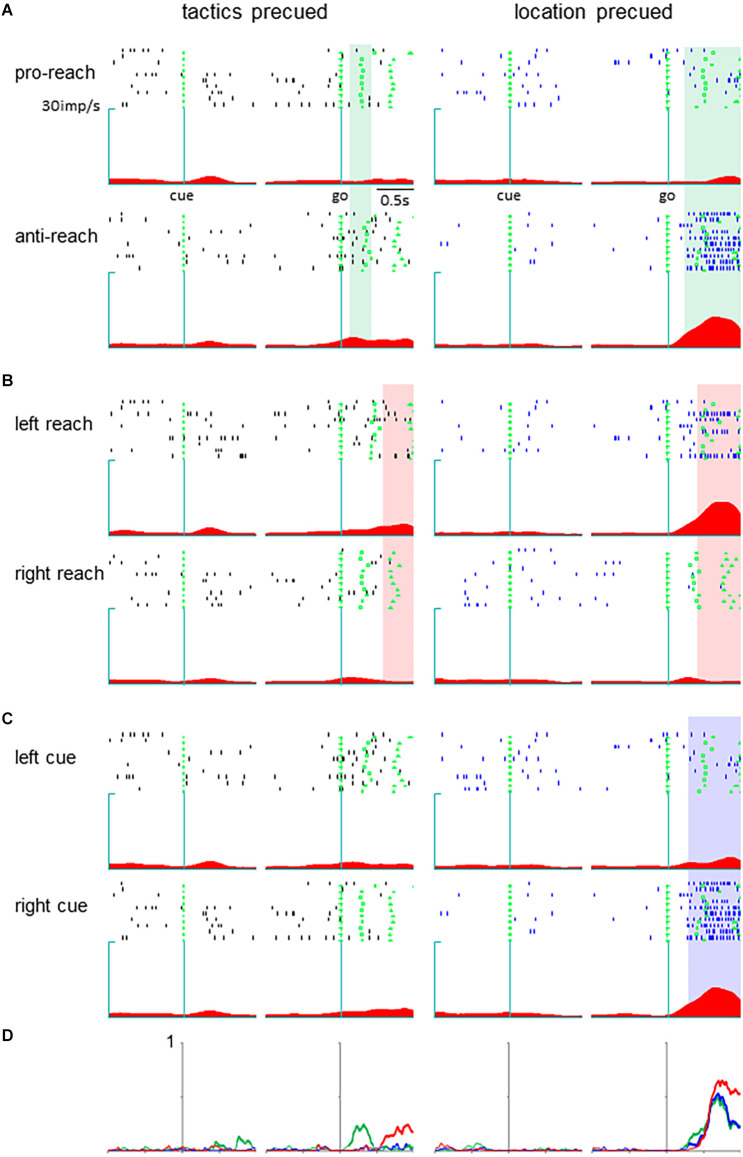
A pmPFC neuron encoding different information during the response period of the two tasks. The legends are the same as in Figure 2.

Neurons in the pmPFC exhibited selective activity for the response determinants or the monkey’s action in various task periods in either the tactics or location-precued tasks. Among them, we found a group of neurons that encoded different parameters during the response period, where the same set of information was given to the monkey in both the tactics and the location-precued tasks. Shown in Figure 2 is a representative example of such pmPFC neurons. In the tactics-precued task, this neuron exhibited selective activation for the tactics, cue location and the monkey’s impending action following the go signal. In contrast, in the location-precued task, it exhibited selective activation for the action while its activity was non-selective for the tactics and the cue’s location. Another group of neurons exhibited more multiplexed representation during the response period of the location-precued task than the tactics-precued task (Figure 3).

**Figure 4:**
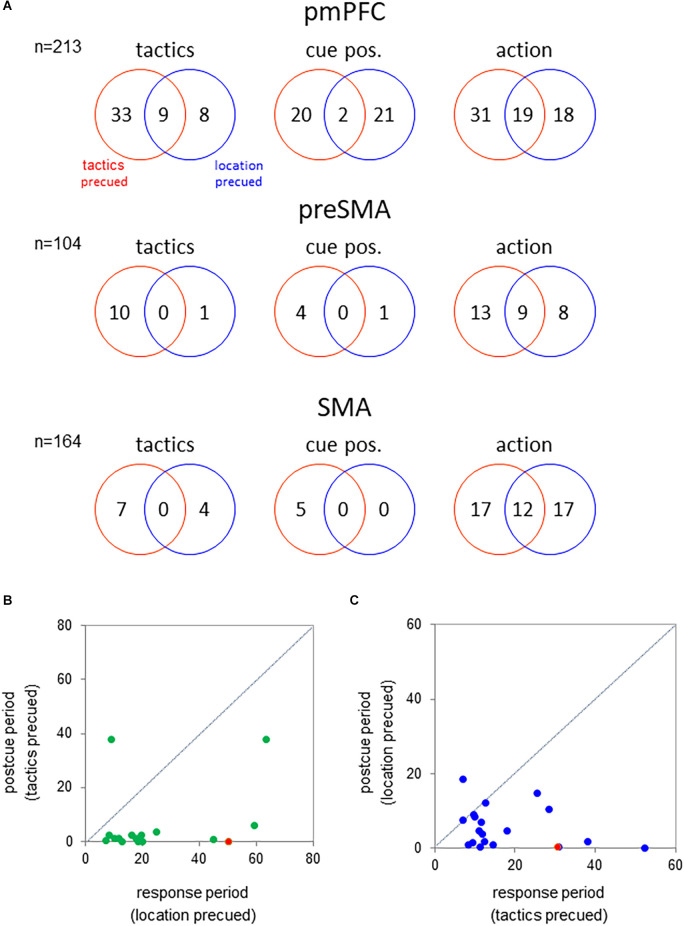
**(A)** Number of neurons showing significant effect of tactics (left), cue position (middle), and action (right) on the activity during the response period (300 ms before hold release) of the tactics- and location-precued tasks (*p* < 0.01 by multifactorial ANOVA). *Top*—pmPFC; *middle*—pre-SMA; *bottom*—SMA. **(B)** Comparison of the tactics-selectivity of the neuronal activity following the onset of the tactics cue between the different task periods. *abcissa*—*f*-value of the multifactorial ANOVA using the tactics as the factor during the response period of the location-precued task; *ordinate*—*f*-value during the post-cue period of the tactics-precued task. The green circles represent the pmPFC neurons that encoded tactics during the response period of the location-precued task. The red circle represents the neuron in Figure 3. The mean value during the response period was significantly greater than that in the post-cue period (*p* < 0.001 by the two-tailed Kolmogorov-Smirnov test). **(C)** Comparison of the location-selectivity of the neuronal activity following the onset of the tactics cue between the different task periods. Legends are the same as in B except that the abscissa and the ordinate are the *f*-values of the ANOVA using the cue location as the factor. *Blue circles*—pmPFC neurons that encoded the location during the response period of the tactics-precued task; *Red circle*—the pmPFC neuron in Figure 2. The mean value was significantly greater in the response than in the post-cue period (*p* < 0.001 by two-tailed Kolmogorov-Smirnov test).

To examine whether the medial cortical neurons encoded the tactics, cue position, or action either selectively or universally across the behavioral tasks, we analyzed the neuronal representations of these factors in each task separately. For this purpose, we examined their effects on the activity during the response period where all of the information was available to the monkey (multifactorial ANOVA, *p* < 0.01; Figure 4A). In the pmPFC, a total of 50 neurons exhibited tactics-selective activation during the response period. Of them, 41 neurons exhibited such a selective activity in either the tactics- or the location-precued tasks but not in both. Additionally, 43 pmPFC neurons were selectively activated in trials in which the visuospatial cue appeared either the left or the right position. The cue-position representation of these neurons was strongly dependent on the behavioral task. 20 and 21 neurons encoded the cue position exclusively in the tactics- and location-precued tasks, respectively. Only two neurons exhibited the cue position selective activity in both of the tasks. And finally, 70% (49/68) of action-selective neurons exhibited task-dependent representation of the monkey’s impending reach direction.

To examine whether the task-dependent representation of the tactics during the response period was merely a sensory response to the onset of the tactics cue in the location-precued task, we compared the neuronal activity during the response period of the location-precued task and the post-cue period of the tactics-precued task. The majority (16/17) of neurons that encoded tactics during the response period of the location-precued task had reduced selectivity for the tactics during the post-cue period of the tactics-precued task (Figure 4B). Similarly, over half (17/22) of the neurons that exibited location-selective activity selectively in the response period of the tactics-precued tasks were not as location-selective in the post-cue period of the location-precued task (Figure 4C).

Finally, we examined the effect of task-type, action, cue position, and tactics during the response period in the three medial frontal areas (Supplementary Figure S2). In the pmPFC, 84 neurons (40% of all of the selective neurons) showed selectivity for the task-type compared to 35% and 26% of neurons in the pre-SMA and SMA, respectively. In some cases, the neuronal representation of the task-type appeared even before the cue onset, indicating that the monkey anticipated which task it was going to perform (Supplementary Figure S3). The proportion of tactics-selective neurons was also significantly greater in the pmPFC than in the pre-SMA and SMA (23% vs. 5% vs. 3%, respectively). Cue location-selective neurons were most prevalent in the pmPFC (12%) compared to the pre-SMA (2%) and SMA (2%). The percentage of action-selective was comparable among the three areas.

## Discussion

The present study showed that a single neuron can play different roles across different tasks even though the tasks require the same information for execution. In one task, the tactics was cued prior to the visuospatial cue while in the other task, the order of their presentation was reversed. Yet both tasks required the common response determinants (the cue location and the tactics) to decide the monkey’s action. Our results indicate that neurons in this region encode different aspects of task performance even if common information is given in various tasks (Figures 2, 3, and 4).

Neurons in the dorsolateral prefrontal cortex have been known to alter the representation of the same sensory stimuli depending on what task they were used for. Such task-dependent encoding could underly the mechanism by which a limited number of neurons can generate an almost infinite number of rule-based behaviors (Mansouri et al., 2006). For example, Asaad et al. (2000) studied the neuronal activity in the dorsolateral prefrontal cortex (dlPFC) of monkeys while they were alternating among multiple types of two-choice saccade tasks. In their study, a shape cue served either as a sample stimulus of a DMS task or as a conditional stimulus to evoke an associated oculomotor response. The neurons were divided into three groups. One group of neurons encoded stimulus identity regardless of the task-type in which it was presented. Another group of neurons exhibited activity modulation by the type of the task but not by the stimuli. Finally, there was a group of neurons the activity of which was modulated both by the stimulus identity and the task-type. Interestingly, more than 30% of the neurons were purely selective for either the stimulus or the task-type. On the other hand, a relatively small proportion of neurons (14.8%) had mixed effects of stimulus and task. Warden and Miller (2010) compared the neuronal activity across two similar working memory tasks designated as “recognition” and “recall.” They found that task context has a profound influence on neural selectivity. The percentage of neurons that were activated in both tasks was quite similar to the percentage activated in one task (49% vs. 51%, respectively).

In our study, more than half (16/25 = 64%) of the cue location-selective neurons in the pmPFC exhibited a significant effect of the task (Supplementary Figure S2). Similarly, the number of task-dependent stimulus-selective neurons slightly exceeded that of task-independent stimulus-selective neurons in the lPFC (Asaad et al., 2000). Further, the majority of the cue location-selective neurons had selective activity in only one task (Supplementary Figure S2). This is consistent with findings in the lPFC (Warden and Miller, 2010). In addition to the neuronal representation of the stimulus properties, the present study indicates that the task-type had a profound influence also on the neuronal representations of the tactics inferred from the stimuli and the resultant action (Supplementary Figure S2).

The prefrontal cortex is involved in logical information processing, leading to flexible behavior (Blackman et al., 2016). Mante et al. (2013) reported that the prefrontal cortex is a dynamic system where context-dependent selectivity for relevant stimulus component is implemented by separate neuronal populations. Particularly relevant to this issue is the discovery that, after training on various tasks, prefrontal neurons showed decreased trial-to-trial variability of discharges and noise correlation between neurons so that new information is incorporated into the activity of a small population of neurons (Qi and Constantinidis, 2013; Tang et al., 2022). In most of these studies, neurons were recorded from the lPFC, while we recorded from the medial frontal area. The decorrelation would increase the potential of neural circuits to hold information and thus could enable the coexistence of representations of multiple tasks in the same neural circuit. A limitation of this study is that the rule to determine the action was not fundamentally different between the two tasks. Though reversing the order of the presentations of the cues impacted the encoding of task-relevant variables (Figures 2, 3), further studies are needed regarding the basis of flexible encoding by prefrontal neurons.

The task-dependent encoding of response-determinants and actions in the pmPFC can be explained by the strong coupling between the mPFC and lPFC and the rostral-caudal hierarchical difference. Both regions are densely connected (Barbas and Pandya, 1989) and likely to interact to support cognitive functions (Matsumoto and Tanaka, 2004). Alexander and Womelsdorf (2021) proposed a computational model in which in the early stages of trial neuronal activity in the lPFC developed ahead of the mPFC, and the lPFC modulated activity in the mPFC. The lPFC is involved in contextual control by encoding the current context and sending information to the mPFC. The mid-dlPFC may be at the top of the frontal hierarchy (Badre and Nee, 2018). The output from the lPFC to the mPFC may contribute to the presence of task-dependent encoding response determinants found in the pmPFC.

At the single-neuron level, mPFC neurons show mixed selectivity for task-relevant aspects (Rigotti et al., 2013). These results are consistent with those from our previous study (Awan et al., 2020) where single neurons encoded multiple task-relevant information, such as tactics, action, or cue position, in a mixed manner. In addition, our results indicate that neurons subserve different functions across different tasks, even when they necessitate the utilization of common information to decide the behavioral response. Task-specific coding and task-dependent changes in functions in mPFC neurons in our findings are contrasted with recent findings which focused on more generalized scheme-like neuronal representations across tasks (e.g., Samborska et al., 2022; Flesch et al., 2022; Berners-Lee et al., 2022). Luk and Wallis (2009) proposed a role for the mPFC in encoding information related to the task and the presence of separate neuronal populations encoding the response and outcome.

Taken together, our results show that the same functions could be subserved by different neuronal populations in different tasks. The pmPFC neurons flexibly switch their functions across various tasks, which could enable the performance of diverse rule-guided behaviors.

## Data availability statement

The raw data supporting the conclusions of this article will be made available by the authors, without undue reservation.

## Ethics statement

The study involving animals was reviewed and approved by the Institutional Animal Care and Ethics Committee of the Center for Laboratory Animal Research of Tohoku University.

## Author contributions

YM and MA conceived the study, designed the experiment, and collected the data. MA and YM conducted data analysis and wrote the manuscript. HM contributed to the revision of the manuscript. All the authors approved the submitted version.

## Supplementary material

SUPPLEMENTARY FIGURE S1 Left. Locations of the posterior medial prefrontal cortex (pmPFC), presupplementary motor area (pre-SMA), and the supplementary motor area (SMA). *Middle*: distribution of one monkey’s task-related neurons. The size of the filled circles represents the numbers recorded in each electrode penetration, and the cross signs represent the penetrations where no task-related neurons were recorded. *Right*: sensory responses in each electrode penetration. A, arm; D, digits; E, elbow; FA, face; FT, foot; H, hand; K, knee; F, Flank, L, leg; SH, shoulder; and V, visual.

SUPPLEMENTARY FIGURE S2 Venn diagrams of the numbers of selective neurons in the pmPFC (top), pre-SMA (middle), and SMA (bottom). The effects of the tactics (pro- vs. anti-reach), cue position (left vs. right), action (reaching to left vs. right), and task type (tactics- vs. location-precued) on neuronal activity during the response period (300 ms time interval prior to hold release) were analyzed by multifactorial ANOVA. The criterion for a significant effect was *p* < 0.01.

SUPPLEMENTARY FIGURE S3 A pmPFC neuron which encoded the task-type throughout the entire trial. **(A–C)** Same legends as in Figures 2A–C. **(D)** The temporal variance of neuronal selectivity for tactics (green), cue position (blue), action (red), and the task type (brown). Data from both tasks were combined to compute the coefficients of partial determination (CPD) of these factors. Thick lines indicate a significant effect of the corresponding factor on IFR(*t*) (*p* < 0.05 by ANOVA).
