# Supplementary figures and images for: Non-overlapping sets of neurons encode behavioral response determinants across different tasks in the posterior medial prefrontal cortex
Source: Front Syst Neurosci. 2023 Feb 9;17:1049062. doi: 10.3389/fnsys.2023.1049062 (PMC9947505; doi:10.3389/fnsys.2023.1049062)

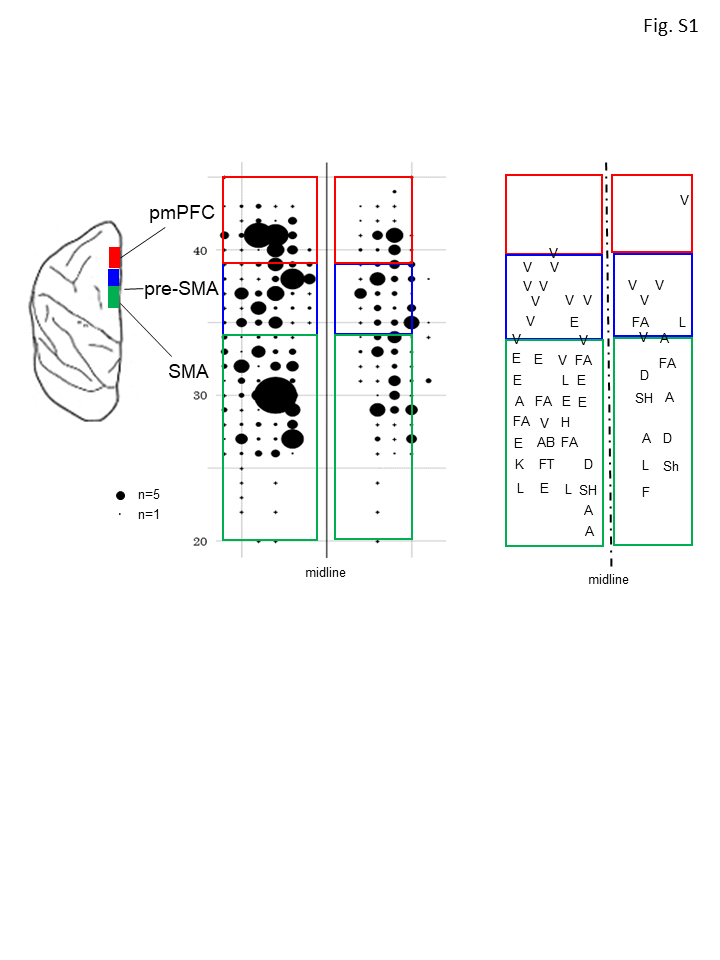

Supplement: SUPPLEMENTARY FIGURE S1 — Left. Locations of the posterior medial prefrontal cortex (pmPFC), presupplementary motor area (pre-SMA), and the supplementary motor area (SMA). Middle: distribution of one monkey’s task-related neurons. The size of the filled circles represents the numbers recorded in each electrode penetration, and the cross signs represent the penetrations where no task-related neurons were recorded. Right: sensory responses in each electrode penetration. A, arm; D, digits; E, elbow; FA, face; FT, foot; H, hand; K, knee; F, Flank, L, leg; SH, shoulder; and V, visual. [file Image_1.TIF]

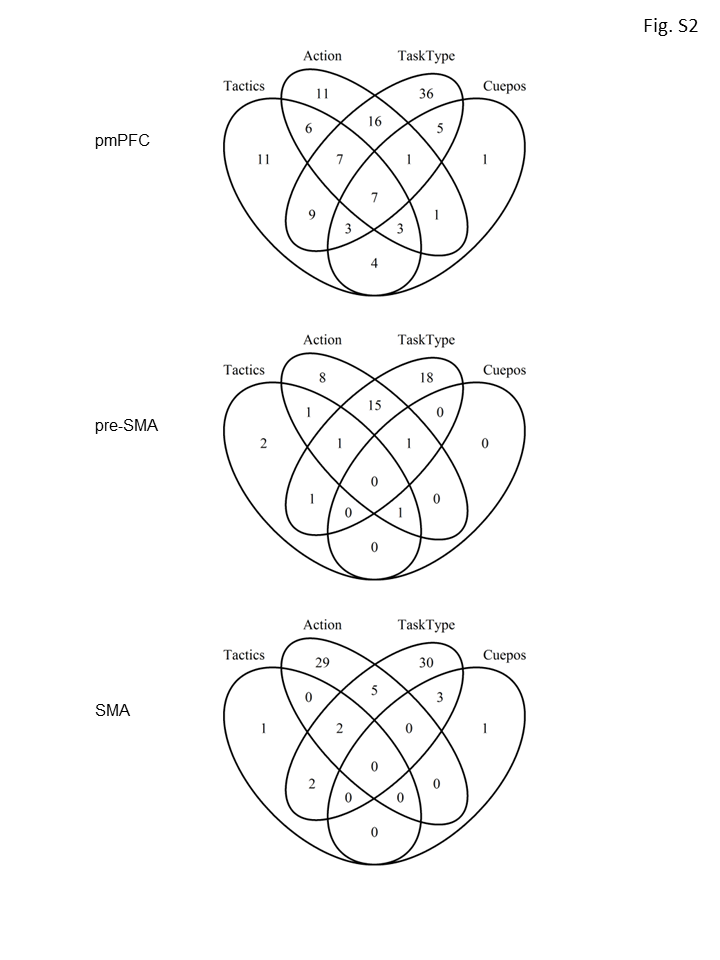

Supplement: SUPPLEMENTARY FIGURE S2 — Venn diagrams of the numbers of selective neurons in the pmPFC (top), pre-SMA (middle), and SMA (bottom). The effects of the tactics (pro- vs. anti-reach), cue position (left vs. right), action (reaching to left vs. right), and task type (tactics- vs. location-precued) on neuronal activity during the response period (300 ms time interval prior to hold release) were analyzed by multifactorial ANOVA. The criterion for a significant effect was p < 0.01. [file Image_2.TIF]

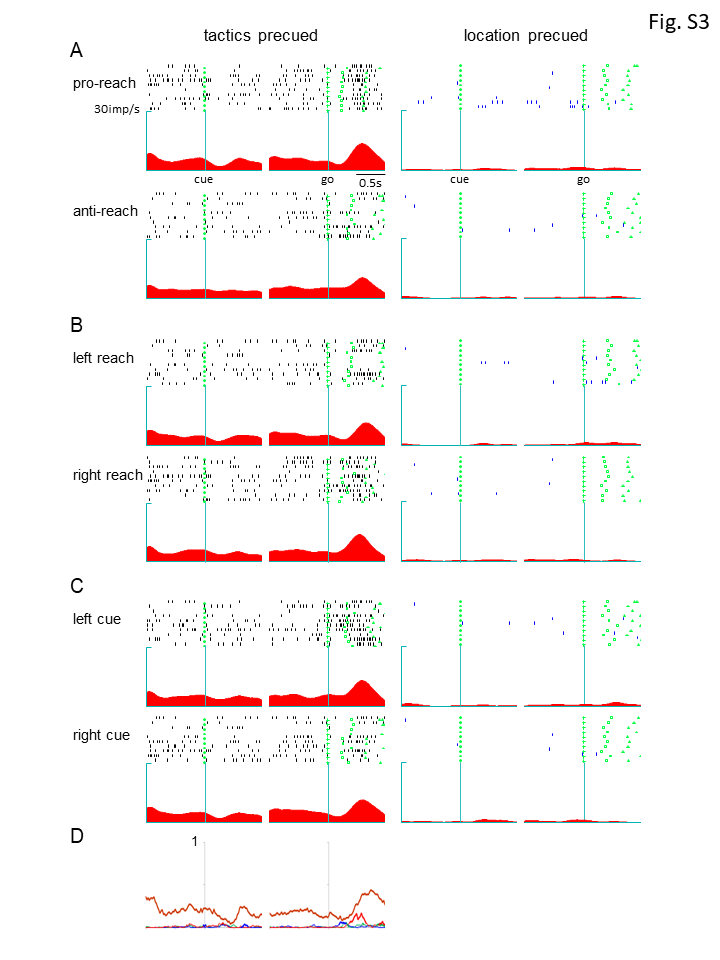

Supplement: SUPPLEMENTARY FIGURE S3 — A pmPFC neuron which encoded the task-type throughout the entire trial. (A–C) Same legends as in Figures 2A–C. (D) The temporal variance of neuronal selectivity for tactics (green), cue position (blue), action (red), and the task type (brown). Data from both tasks were combined to compute the coefficients of partial determination (CPD) of these factors. Thick lines indicate a significant effect of the corresponding factor on IFR(t) (p < 0.05 by ANOVA). [file Image_3.TIF]
